# Therapeutic hypothermia for acute brain injuries

**DOI:** 10.1186/s13049-015-0121-3

**Published:** 2015-06-05

**Authors:** Max Andresen, Jose Tomás Gazmuri, Arnaldo Marín, Tomas Regueira, Maximiliano Rovegno

**Affiliations:** Departamento de Medicina Intensiva, Facultad de Medicina, Pontificia Universidad Católica de Chile, Marcoleta, 367 Santiago Chile; Hospital de Urgencia Asistencia Pública, Facultad de Medicina, Pontificia Universidad Católica de Chile, Santiago, Chile

**Keywords:** Hypothermia, Target temperature management, Cardiac arrest and brain injuries

## Abstract

Therapeutic hypothermia, recently termed target temperature management (TTM), is the cornerstone of neuroprotective strategy. Dating to the pioneer works of Fay, nearly 75 years of basic and clinical evidence support its therapeutic value. Although hypothermia decreases the metabolic rate to restore the supply and demand of O_2_, it has other tissue-specific effects, such as decreasing excitotoxicity, limiting inflammation, preventing ATP depletion, reducing free radical production and also intracellular calcium overload to avoid apoptosis. Currently, mild hypothermia (33°C) has become a standard in post-resuscitative care and perinatal asphyxia. However, evidence indicates that hypothermia could be useful in neurologic injuries, such as stroke, subarachnoid hemorrhage and traumatic brain injury. In this review, we discuss the basic and clinical evidence supporting the use of TTM in critical care for acute brain injury that extends beyond care after cardiac arrest, such as for ischemic and hemorrhagic strokes, subarachnoid hemorrhage, and traumatic brain injury. We review the historical perspectives of TTM, provide an overview of the techniques and protocols and the pathophysiologic consequences of hypothermia. In addition, we include our experience of managing patients with acute brain injuries treated using endovascular hypothermia.

## Review

### History

Therapeutic hypothermia for acute brain injury is the intentional lowering of body temperature, with the objective of reducing tissue damage in the central nervous system. Modern use of therapeutic hypothermia as a neuroprotective strategy began in the 1940s with the work of Fay [1], who reported the first series of patients with traumatic brain injury who were treated using hypothermia. Therapeutic hypothermia has become a standardized method of care for improving neurological results after cardiac arrest. This approach has been used since the publication of two randomized studies with positive outcomes, each of which used 33°C for 12 h to 24 h [2,3], respectively. Recently, a new randomized trial revisited the question of target temperature and was unable to demonstrate any difference in mortality or in adverse neurologic outcome in patients treated at 33°C compared with those treated at 36°C [4]. Therapeutic hypothermia has been historically classified into: mild (34.5–36.5°C), moderate (34.5–32°C), marked (28–32°C) and profound hypothermia (<28°C) [5,6]. Actually, there is a recommendation from five professional societies replaced the term “therapeutic hypothermia” with the term “target temperature management” (TTM) [7], which implies a broad range for controlling body temperature; TTM can also be applied to normothermia using a similar approach.

### Overview of TTM

Techniques to induce and maintain hypothermia can be divided into two types: external and internal cooling methods. The first type includes the use of cooling blankets, ice packs, alcohol baths, cold-water immersion, cold-saline gastric lavage, and local cooling using helmet devices. However, despite their non-invasive nature, these methods have some disadvantages, such as complex implementation, particularly in obese patients, high nursing requirements, intense skin vasoconstriction - shivering, slow onset of the desired temperature and erratic temperature maintenance [8]. Nevertherless, other surface cooling devices allow heat exchange by external water circulating, and using automatic feedback-control temperature mechanism. Following cardiac, it arrest appears safe and effective in maintaining the target body temperature, with lesser variation than traditional cooling blankets [9,10].

The second type includes internal cooling methods that use central venous catheters to either infuse cool saline or directly to reduce the blood temperature by convection. A single study compared the cooling rates for external and internal methods, showing rates of 0.9°C/h and 4.8°C/h, respectively [11].

**Figure 1:**
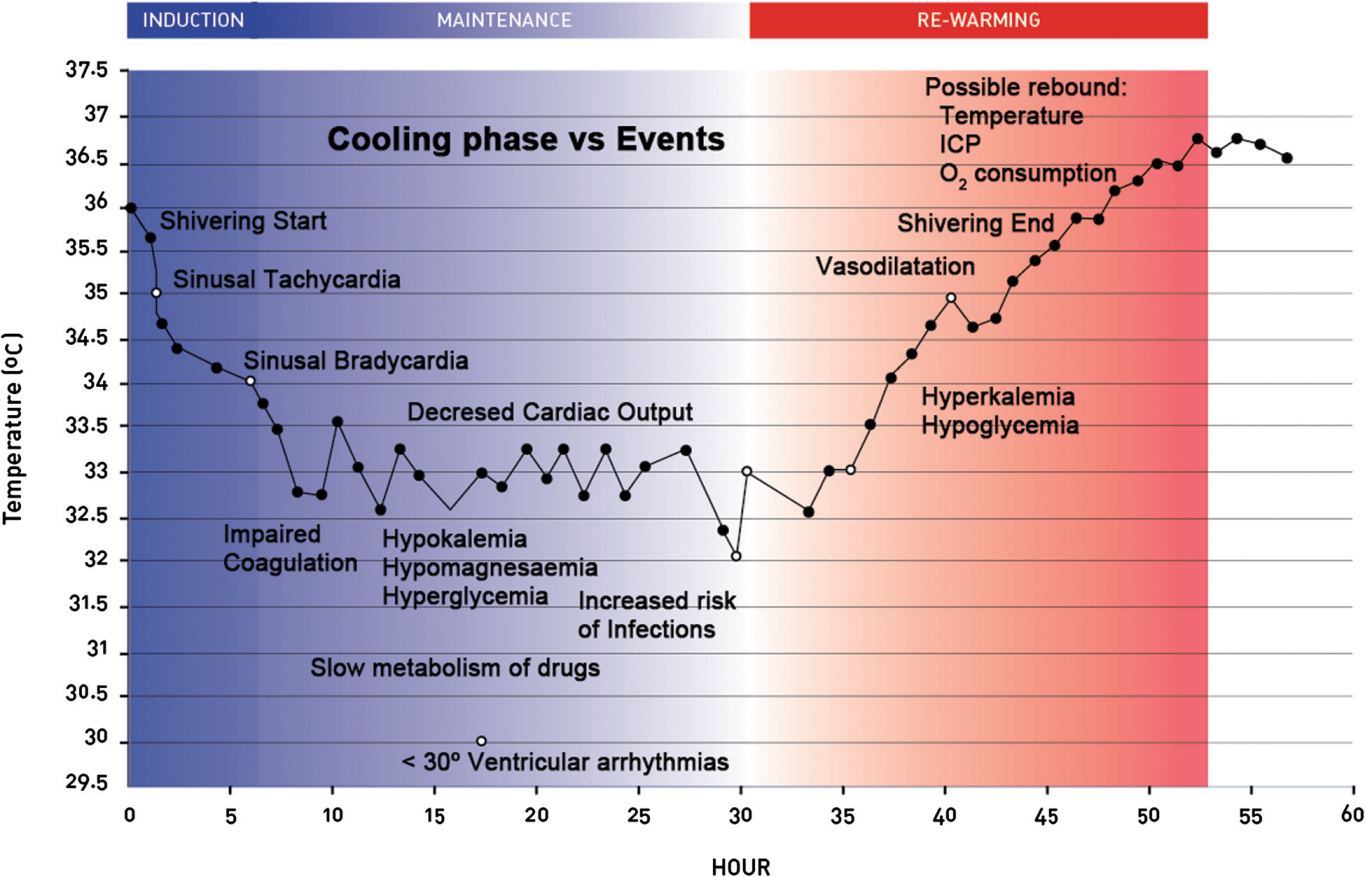
Physiological changes during hypothermia. Severe specific events may occur during the three phases of hypothermia procedure. Time scale in hours is for illustrative purposes only.

Induced hypothermia can be separated into three phases: induction, maintenance and rewarming (Figure 1), each phase produces several changes in normal physiology. Clinicians should be aware of phase events and inter individual differences. Hypothermia induction should be started as soon as possible to minimize neurologic damage. Infusing cold fluids, e.g., Ringer’s lactate >25 ml/kg at 4°C, is the easiest and most effective method for inducing hypothermia [12]. During hypothermia, the patient’s central temperature should be closely monitored. The preferred probe locations are brain or core temperature, such as central venous, esophageal, tympanic, nasopharyngeal, bladder and rectum sites. This order represents the grade of correlation with brain temperature [13-15].

Hypothermia is not exempt from complications (Figure 1). Major hypothermia-related complications include cardiac arrhythmias, coagulopathy, hypokalemia, and infections, of which pneumonia is the most frequently reported [16,17]. Other reported minor complications are thrombocytopenia, hyperamylasemia, prolonged PR and QT intervals and sinus bradycardia, which are generally not associated with important clinical impact [16]. Currently, these complications are not common, because most of them develop with temperatures of 32°C and current protocols use target temperatures of 33–34°C. Lower temperature levels have not demonstrated benefits [18]. Internal cooling methods are associated with vascular complications, such as catheter-related infections, deep venous thrombosis and vascular dissection. Fortunately, these types of complications occur in <4% of cases [9,19].

A key adverse effect of hypothermia is shivering, which may cause great discomfort to the patient, triggering massive increases in systemic and cerebral energy consumption, that produce considerably slower cooling rates and increase intracranial pressure (ICP) [20-22]. Previously, the use of profound sedation and neuromuscular blockades were the only methods to avoid shivering. However, two studies have demonstrated that it is possible to maintain mild hypothermia with conscious patients using a combination of non-pharmacologic approaches, such as external warming (available for internal cooling methods) and low doses of meperidine and buspirone [23,24].

### Hypothermia and acute brain injury

#### Ischemic injury: stroke and cardiac arrest

Currently, stroke is a prevalent cause of morbimortality worldwide. Death and severe functional impairment rates remain high, because of the low likelihood of timely thrombolysis treatment. Nearly 70% of acute stroke patients arrive in the emergency room at a time that exceeds the therapeutic window for using thrombolysis. The door-to-needle times account for another percentage of these high rates [25]. Finally, fewer than 5% of the candidate patients receive thrombolysis [26].

Numerous studies of stroke in animal models have evaluated the benefits of therapeutic hypothermia. Some of them have demonstrated the efficacy of therapeutic hypothermia before or during experimental brain ischemia; however, they lack clinical applicability because these scenarios do not represent real life (Table 1). One meta-analysis of these data concluded that hypothermia reduced approximately 44% of the infarction size and improved the neurological outcome; moderate hypothermia was required to obtain this benefit, although it could also be achieved with mild hypothermia. A shorter elapsed time from injury to hypothermia resulted in a smaller infarction size [27].

**Table 1:** **Summary of therapeutic hypothermia; indications, performance, type of evidence and proposed protocols**

**Clinical Scenario**	**Efficacy**	**Evidence**	**Protocol**
Cardiac arrest (VT or VF)	Effective	2 small RCTs and multiple cohort studies	Temperature target 32-34°C for 12–24 h [2,3,30,31]
Neonatal hypoxic ischemic encephalopathy (HIE)	Effective	RCTs	Moderate or severe HIE, should be treated within 6 h of delivery to 32–34°C for 72 h, at slow rewarming rate [34,35]
Increased ICP	Effective	RCTs and cohort studies	32–36°C (tailored according ICP level) [17,64,65,68]
Cardiac arrest (PEA or asystole)	Possible	Case series	Target temperature 32–34°C for 12–24 h [9,10,12,69]
Hypoxic encephalopathy in hanging injury cases	Feasible	Case series	Target temperature 32–34°C > 48 h [70,71]
Ischemic Stroke	Feasible	Small RCTs, ongoing trials	35°C for awake patients, 32–35°C for ventilated comatose patients [36-39]
Intracerebral hemorrhage	Unknown	Case series	Fever control [48]
Subarachnoid hemorrhage	Unknown	Case series	Fever Control [52]
Traumatic brain injury	Unknown	RCTs with conflicting research findings, ongoing trials	Target temperature 32–34°C for > 48 h [59-63]

Protective mechanisms underlying hypothermia are related to the reduction of energy consumption by blocking intracellular signaling events, such as calcium movements that prevent ATP depletion and free radical production. Moreover, hypothermia modulates the inflammatory and apoptotic signaling pathways, favoring trophic and anti-apoptotic protein synthesis [28], resulting in a reduction of excitatory neurotransmitters and inflammatory response [29].

Since 2002, when the trial “Hypothermia after Cardiac Arrest Study Group” demonstrated the clinical benefits of therapeutic hypothermia for improving neurological and mortality outcomes in patients suffering TV/FV cardiac arrest [3], many studies have shown the positive effects of hypothermia on neuronal protection in global brain ischemia [2,30,31]. However, the evidence of therapeutic hypothermia for focal cerebral ischemia in strokes remains inconclusive. The disparity in the outcomes may be attributed to the pathophysiological mechanism differences: cardiac arrest is associated with global hypoperfusion, whereas ischemic stroke entails an abrupt and focal interruption of brain circulation, generating an irreversible infarction zone and a peripheral ischemic zone (penumbra), which is capable of recovery. It is important to consider that the neuronal death-associated mechanism in focal cerebral ischemia, primarily occurs through anoxic - ischemic cell death, whereas in global hypoperfusion, there is an injury cascade (such as glutamate excitotoxicity, calcium intracellular intoxication and free-radical injury) that evolves secondary to molecular cell death [32]. Because hypothermia has been demonstrated to play a key role in preventing reperfusion injury, this phenomenon is less important in the focal setting of stroke [32,33]. Indeed, another recognized indication for hypothermia is neonatal encephalopathy, which is a model of global brain ischemia, similar to cardiac arrest, and there is substantial evidence that this treatment reduces the risk of death or disability in infants [34,35].

Whereas nearly every study of therapeutic hypothermia in strokes assumes previous use of thrombolysis, only a few trials have directly studied the safety of using a combination of both therapies [6]. From them, we highlighted the ICTuS-L trial (Intravenous Thrombolysis plus Hypothermia for Acute Treatment of Ischemic Stroke). Unfortunately, this study included a small number of patients (n=59) and was underpowered to demonstrate a significant benefit in the treated group. However, this trial established the feasibility and safety of using both therapies. An increased rate of complications was not reported [36].

Several clinical trials have used hypothermia as a rescue therapy in patients who are considered to be unsuitable for thrombolysis. Schwab et al. showed that hypothermia played a key role in the prevention of brain edema after the thrombolytic window period in hemispheric infarcts. Another study highlighted the importance of a slow rewarming phase to avoid a rebound effect of ICP [37,38]. Moreover, the combination of hypothermia and hemicraniectomy in massive strokes has been tested in small prospective studies. Only, a slight but not statistically significant benefit was demonstrated in terms of neurological outcomes [39].

#### Hemorrhagic injury: stroke

Spontaneous hemorrhagic stroke is associated with greater mortality than is ischemic stroke. There is no specific therapy with proven benefits for hemorrhagic stroke, which can reach a 30-day mortality rate of up to 52% [40]. The prognostic factors for hemorrhagic stroke are the initial size of the hematoma, re-bleeding events, hematoma expansion and peri-hemorrhagic edema [41-43]. Also, an elevated serum glucose measurement at the time of admission is a strong and less well-known poor prognostic factor [44]. It is a stress response marker and is associated with a larger hematoma size, cell death, hematoma expansion and peri-hemorrhagic edema [44,45].

In cases of intraparenchymal hemorrhage, few studies have used hypothermia, showing that hypothermia can reduce the disruption of the blood–brain barrier and peri-hematoma edema. However, these findings have not been correlated with improved neurological outcomes [46,47]. Furthermore, it has been shown that hypothermia during hemorrhagic stroke is not associated with a reduction in the size of the original lesion [47]. Only a small clinical study has suggested that hypothermia has the positive effect of reducing the extent of edema during the first seven days; this effect is not lost with rewarming [48].

A Cochrane review published in 2009, which included eight clinical trials (six randomized and two controlled, n=423) concluded that there is not a significant positive effect of hypothermia in the management of ischemic and hemorrhagic stroke [49].

#### Hemorrhagic injury: SAH

Hyperthermia worsens the neurological evolution of patients suffering ischemic or hemorrhagic stroke. Among hemorrhagic strokes, SAH represents a particular scenario for the usefulness of TTM. These patients exhibit a high rate of hyperthermia, as much as 70% during the first 10 days, been the intraventricular hemorrhage one of the primary risk factors [50].

The use of hypothermia in SAH goes back as far as 1954, when it was used as a neuroprotective measure against bleeding or ischemia during aneurysm repair surgery [51]. Badjatia et al. studied the effects of temperature control and neurologic outcomes in SAH. They demonstrated a reduced risk of poor neurologic outcome 12 months after SAH; however, it should be noted that the studied intervention was the aggressive management of fever versus conventional fever control and not formal hypothermia [52].

The “Intraoperative Hypothermia for Aneurysm Surgery Trial” studied the impact of hypothermia during acute aneurysmal SAH clipping procedures. This multicenter randomized clinical trial (n=1001) demonstrated that hypothermia was associated with more frequent bacteremia episodes, without clinical benefit. This result could be explained because of the good previous clinical condition in both study arms and the absence of an important acute brain injury in the selected patients [53].

#### Traumatic injury: TBI

Traumatic brain injury is the primary cause of worldwide morbimortality in young people (<45 years old) [54]. The annual incidence of TBI in the USA has been estimated to be 1.4 million cases, with 50,000 related deaths [55], whereas in Europe, there is a similar landscape [56]. Today, a specific treatment remains lacking.

Notably, cell damage is not only produced by the trauma itself, but by a series of pathophysiological mechanisms, which increase and perpetuate the damage caused by the initial insult. The mechanisms of this secondary damage are cerebral ischemia and hypoxia, excitotoxicity, inflammation, oxidative stress, metabolic dysfunction, seizures, brain edema and ICP elevation, namely, intracranial hypertension (IH), through diminished brain perfusion pressure. IH is one of major determinant of outcome [57,58].

Evolution of secondary damage mechanisms has been studied in the experimental setting, and can be divided into three distinct phases: acute, sub-acute and chronic. Varying from acute mechanism such as excitotoxicity, intracellular enzyme activation, free radical production and ischemia to subacute inflammation and chronic gliosis formation. This evolution supports an important point: neuroprotective treatments for TBI should be extended up to 72 h.

Marion et al. in a 1997 published a randomized study involving 84 patients with severe TBI who were treated using mild hypothermia (33°C for 24 h). There was a significantly better neurologic recovery at 3 months and at 6 months among the patients with GCS scores of 5–7 at the time of admission to the hospital [59]. At the time of this review, not a single therapy for patients with TBI had demonstrated such powerful neuroprotective efficacy. Subsequent clinical studies have initiated late hypothermia in the course of TBI. Clifton et al. published two of the largest series of patients with severe TBI (n=392 and 232, in 2001 and 2011, respectively, NABIS: H I & II), who were randomized to early hypothermia (2–5 h) versus normothermia. Both studies showed that therapeutic hypothermia had no impact on the neurologic results [60,61].

Jiang et al. revisited the time-dependent effect of hypothermia for TBI. In 2006 published a randomized study of 215 patients with severe TBI, which showed that prolonged hypothermia (5 ± 1.3 days) was more effective than conventional hypothermia (2 ± 0.6 days) in reducing the number of patients with poor neurological outcomes. Both patients arms where cooling to reach 33–35°C of rectal temperature, using cooling blankets [62].

Kramer et al. in a 2009 Cochrane review, included 23 trials and 1,614 patients with the following criteria: early hypothermia, target temperatures of 35°C for at least 12 h and the need for hospitalization. Authors concluded that those patients treated with hypothermia had better results in terms of mortality and neurologic outcomes. However, this association was statistically significant only in studies with poor methodological quality [63].

To prevent ongoing brain edema and refractory IH management, the use of hypothermia in the sub-acute phase has been widely studied. In 2003, Tokutomi et al. presented a series of 31 high-risk patients who were hospitalized for severe TBI with GCS scores of ≤5 at the time of admission to the hospital. Almost, 74% of the patients required surgical management. In addition to show the benefit of the use of hypothermia for treating IH, Tokutomi et al. described an optimal coupling between blood flow and brain metabolism at 35°C, with no compromise in perfusion pressure [64]. The same authors published another trial, using 35°C and compared the results with patients who were previously treated at temperatures of 33°C; the *post hoc* analysis demonstrated better control of brain perfusion pressure at 35°C without differences in ICP levels, complications, mortality or neurological outcomes [65].

In April 2010, the European Society of Intensive Care Medicine started a multicenter randomized clinical trial with the aim of recruiting 1,800 TBI patients to evaluate the benefits of therapeutic hypothermia (32–35°C) in ICP control, morbidity and six-month mortality [66]. Interestingly, this study did not establish common criteria for the duration of hypothermia; however, it was suggested prolonging hypothermia as long as necessary to maintain an ICP of <20 mmHg.

Finally, a systematic review in 2012 collected 13 randomized clinical trials and 5 observational studies concerning IH management in patients with TBI using therapeutic hypothermia. A significant reduction of ICP was evident in all of the patients [17].

### Our experience

In the last decade, our group was inspired by the lack of neuroprotective treatments and by the use of hypothermia after TV/FV cardiac arrest to explore the effectiveness of hypothermia in other clinical settings, such as cardiac arrest due to non-shockable rhythms, acute liver failure with brain edema, refractory intracranial hypertension (rIH) or full neuroprotection intended for acute brain injuries. The medical management of rIH is complex because of the associated unfavorable clinical outcomes [57] and toxic treatment alternatives, such as barbiturate coma [67]. Additional challenges arise from the prolonged nature of IH, which can continue for approximately one week independent of the nature of the acute brain injury. Debate exists concerning the optimal strategies for treating these patients. We studied the performance and outcomes of patients who received therapeutic hypothermia in the last five years.

We defined rIH without surgically correctible causes as ICP >20 mmHg for over one hour, despite a management protocol using the first and second tiers of treatment. We included in a common database all patients who were admitted to our intensive care to undergo therapeutic hypothermia from 2008. For referral reasons, we did not receive patients who were in cardiac arrest due to shockable rhythms. Hypothermia was performed using endovascular cooling (Alsius, Zoll, Chelmsford, MA, USA) with a target temperature of 33°C. Data are expressed as median and interquartile range [IQR].

To date, we have included 30 patients with an average age of 28 years [24–40; IQR], 53% male, and an average APACHE II score of 16 [12–18; IQR]. Of these, 25% of the patients were referred for hypothermia after cardiac arrest, 50% were referred for rIH, and 25% were referred for full neuroprotective intent. The etiology of refractory intracranial hypertension was diverse, including acute brain injury (TBI, malignant stroke, SAH, and post-neurosurgical edema) and liver failure. In patients with rIH, the ICP levels were 23 mmHg [19–24; IQR] before hypothermia versus 13 mmHg [10.3–24; IQR] during hypothermia (p=0.003). Mortality was 36% in this cohort, and the mean Glasgow Outcome Score-Extended (GOS-E) at three months was 5 [3–7; IQR] in the survivors. Cardiac arrest survivors, not VT/FV patients, had poor outcomes with a mean GOS-E of 4 at three months. Notably, the surviving patients with rIH had a lower percentage of time with ICP >20 mmHg during the entire course of hypothermia: 3.3% [0.6–7; IQR] versus 27.2% [19.8–44.9; IQR] (p < 0.01). The complications related to hypothermia did not exceed two per patient, and none required withdrawal of the hypothermia procedure.

Thus, in our experience, hypothermia exhibited modest performance after cardiac arrest of non- shockable rhythms. However, hypothermia was an effective and safe alternative for controlling ICP in patients with rIH, thus helping to produce good neurological outcomes in survivors.

## Conclusions

Hypothermia has evolved as the strongest neuroprotective intervention in clinical therapy. Currently, treatment with hypothermia is standard medical care after VT/VF cardiac arrest-related coma, and neonatal encephalopathy. Its utility, in addition to treatment for cardiac arrest, is noted in the course of acute brain injuries. However, from our perspective, hypothermia can be used with a reasonable likelihood of success when IH complicates any brain injury, such as stroke or traumatic brain injury, because IH diminishes the brain blood pressure, generating a condition recognized as global brain hypoperfusion. In this situation, the pathophysiological mechanisms of brain injury are susceptible to treatment with hypothermia. The primary objective of hypothermia is to preserve the central nervous system tissue while making time to resolve the original pathology. Although, this recommendation represents only the beginning of TTR because there are many details that remain to be elucidated, such as the exact time window for each pathology, the time of treatment, the target temperature, rewarming protocols, adequate therapeutic markers and what therapies in combination with hypothermia would offer better neuroprotection.

## Abbreviations

ICP: Increase intracranial pressure
IH: Intracranial hypertension
IQR: Interquartile range
rIH: Refractory intracranial hypertension
SAH: Subarachnoid hemorrhage
TBI: Traumatic brain injury
TTM: Target temperature management
TV/FV: Tachycardia ventricular/fibrillation ventricular
